# Representations of uncertainty: where art thou?

**DOI:** 10.1016/j.cobeha.2021.03.009

**Published:** 2021-04

**Authors:** Ádám Koblinger, József Fiser, Máté Lengyel

**Affiliations:** 1 Center for Cognitive Computation, Department of Cognitive Science, Central European University, Hungary; 2 Computational and Biological Learning Lab, Department of Engineering, University of Cambridge, United Kingdom

## Abstract

Perception is often described as probabilistic inference requiring an internal representation of uncertainty. However, it is unknown whether uncertainty is represented in a task-dependent manner, solely at the level of decisions, or in a fully Bayesian manner, across the entire perceptual pathway. To address this question, we first codify and evaluate the possible strategies the brain might use to represent uncertainty, and highlight the normative advantages of fully Bayesian representations. In such representations, uncertainty information is explicitly represented at all stages of processing, including early sensory areas, allowing for flexible and efficient computations in a wide variety of situations. Next, we critically review neural and behavioral evidence about the representation of uncertainty in the brain agreeing with fully Bayesian representations. We argue that sufficient behavioral evidence for fully Bayesian representations is lacking and suggest experimental approaches for demonstrating the existence of multivariate posterior distributions along the perceptual pathway.

**Current Opinion in Behavioral Sciences** 2021, **38**:150–162

This review comes from a themed issue on **Computational cognitive neuroscience**

Edited by **Angela J Langdon and Geoffrey Schoenbaum**

For a complete overview see the Issue and the Editorial

Available online 1st April 2021

**https://doi.org/10.1016/j.cobeha.2021.03.009**

2352-1546/© 2021 The Authors. Published by Elsevier Ltd. This is an open access article under the CC BY license (http://creativecommons.org/licenses/by/4.0/).

## Introduction

In order to efficiently interact with our environment, we need to evaluate the potential consequences of our decisions. Critically, these decisions are usually based on information that is limited and ambiguous in several ways. For example, when we choose where to look for our bicycle at the parking station at the end of the day, we are coping with occlusions by other similar bikes, low visibility, and incomplete memories about where our bike was left when we came to work. Therefore, in general, we cannot know with certainty the values of those variables that are relevant to our decisions, and optimal decision making requires that we take this uncertainty into account.

Indeed, a large body of evidence indicates that humans, and other animals, make decisions by representing their uncertainty [1, 2, 3, 4]. However, it remains unclear how general a computational strategy it is for the brain to represent and compute with uncertainty in complex environments characterized by many interacting variables (i.e. arguably just about any real-life scenario). In particular, it is largely unknown whether the brain represents uncertainty ‘opportunistically’, only about the variables that are relevant for the decision at hand, or ‘constitutively’, about many variables simultaneously, including ones that are not directly relevant for the current decision making situation. In the context of our example above, the opportunistic strategy would only represent uncertainty about the single high-level decision variable (the location of the bike relative to where we stand). In contrast, the constitutive strategy would represent uncertainties about several perceptual and other variables that feed into the decision process, such as the reliability of perceived color in the darkness, the ambiguity of shape information given partial occlusions within the crowd of bikes, and the precision of our memories about the layout of the parking station.

The distinction between opportunistic and constitutive representations of uncertainty has not been explicitly articulated before and is therefore the main focus of this review. We begin by building on the classical framework of Bayesian decision theory [5] to formalize the distinction between these representational strategies as task-dependent and fully Bayesian recognition models, evaluate their respective theoretical advantages and disadvantages, as well as the empirical evidence that may be interpreted as supporting them. We also explore different hybrid solutions between these two extreme representational strategies, and discuss the consequences of each of them for the neural representation of uncertainty. We then argue that current evidence is insufficient to clearly distinguish between these different strategies, and propose experimental approaches that are appropriate for identifying their behavioral signatures.

## Four reasons to represent uncertainty

**Figure 1:**
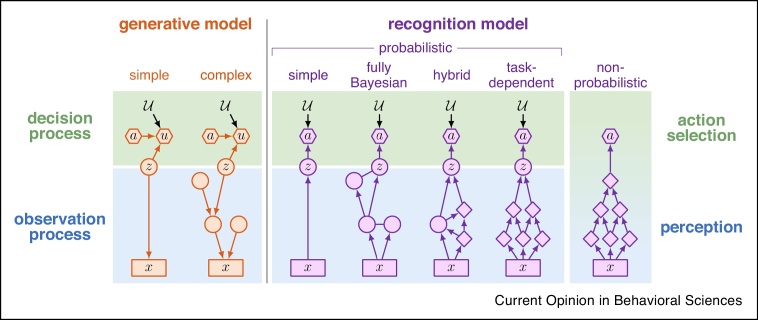
Taxonomy of generative and recognition models in decision making. Blue and green backgrounds correspond to the two components of Bayesian decision theory: the observation and decision processes of the generative model (orange), and the perception and action selection modules of the recognition model (purple), respectively. Note that ‘perception’ here is broadly construed to include all cognitive processes (e.g. sensory perception or memory) that have access to information (‘observations’) that is relevant for the decision making task. Rectangles indicate observed variables (x), circles indicate latent variables (including the decision variable, z) which are part of the generative model and are probabilistically computed in a recognition model, diamonds indicate non-probabilistically computed internal variables of a recognition models, hexagons indicate variables specific to the decision process: the action (a) and the utility obtained (u). The utility function (U) is shown without a bounding box to indicate that it is a parameter that is constant across trials or time steps, while other quantities change over time or trials. Left: generative models. All generative models describe how z is related to x, and how it (exclusively) determines the u obtained for a given a (as parameterized by U). **Simple generative models** only have a single latent variable, z. **Complex generative models** have multiple latent variables beside z. Right: recognition models. All recognition models compute action a from observations x. **Probabilistic recognition models** compute a posterior over z given x (Equation 1), which they then combine with U to compute a (Equations 2a and 2b). The **non-probabilistic recognition model** computes a directly from x, without computing a posterior over z, and without explicitly representing U. Probabilistic recognition models are further subdivided based on what other variables are computed probabilistically while computing the posterior over z: **simple models** do not have any other variables, complex models do, with **fully Bayesian**, **hybrid**, and **task-dependent models** probabilistically computing all variables, a subset of them, or none of them, respectively.

In order to understand the distinction between opportunistic and constitutive representations of uncertainty, we first take a step back, and briefly discuss why representing uncertainty at all is relevant for decision making in the first place. The mathematical framework of Bayesian decision theory provides an answer to this question [5] (Box 1). In this framework, the problem of optimal decision making — and the role of uncertainty in it — can be formalized by defining a relationship between a handful of key variables in the observer's internal model of the decision making situation (Figure 1, 1st column): the decision variable (in our running example: expressing the location of the bicycle relative to where we stand now), the utility (time spent searching for the bike), the action (turning right or left, or going straight), and the observation (current visual input as well as the memory traces stored from the time when we left the bike at the station). The specific decision making task is ultimately defined by the utility function (also called the reward or loss function) that determines how the utility obtained depends on the decision variable and the action taken. The internal model that defines the relationships between all these ingredients is also called a *generative model* because it describes the observer's beliefs about how the world generates observations and utilities (the latter contingent upon their own actions).

Box 1 **Bayesian decision theory**

According to the agent's internal model (Figure 1, 1st column), there is a decision variable, z, of which the value determines how the utility, u, depends on the different actions they might choose, a (decision process). The exact form of this dependence is determined by the utility function $u=U\left(a,z\right)$. However, z is not directly accessible to the agent, hence it is a *latent variable*. Instead, the agent makes *observations*, x, that refer to all the information that are available to them at the time of making the decision (observation process). Importantly, the agent must also have some knowledge about the (potentially noisy and ambiguous) relationship between z and x (Figure 1, 1st column, arrow connecting z and x).

Given these ingredients, Bayesian decision theory proceeds in two steps to compute the optimal choice of a. First, the value of z needs to be inferred based on x. As z cannot be determined with certainty, Bayes’ rule is used to compute a posterior distribution that expresses the probability with which z might take any particular value given the information in x:

(1) $$P\left(z|x\right)=\frac{P\left(x|z\right)\;P\left(z\right)}{\int P\left(x|z^{\prime }\right)\;P\left(z^{\prime }\right)\;dz^{\prime }}$$

where $P\left(x|z\right)$ (the *likelihood*) expresses the probability of observing x when z takes on a particular value, and $P\left(z\right)$ (the *prior*) expresses the overall frequency with which z is believed to take on any particular value. Second, the utility expected from choosing each action is obtained by considering all possible settings of z, computing the corresponding utilities and then taking the average of these utilities weighted by the corresponding posterior probabilities:

(2a) $$\bar{U}\left(a,x\right)=\int U\left(a,z\right)\;P\left(z|x\right)\;dz$$

The action with the maximal expected utility is chosen as

(2b) $$a\left(x\right)\;\;=\underset{a^{\prime }}{\mathrm{argmax}}\bar{U}\left(a^{\prime },x\right)$$

Alt-text: Box 1

Critically, as the decision variable itself is not observed directly (it is a *latent variable*), its value can only be inferred (based on the observations), and this inference usually carries uncertainty. This uncertainty is formalized by the Bayesian posterior as the probability that the decision variable might take any particular value given the information in the observations (Equation 1). Thus, computing the optimal action under a given internal model requires (1) computing the Bayesian posterior over the decision variable, and then (2) computing the expected utility of each available action under this posterior (Equation 2a). The optimal action is simply the one that yields the highest expected utility (Equation 2b). Straightforward this description may sound, the corresponding computation is anything but: in fact, both steps have prohibitive computational costs in the general case. Borrowing (and slightly extending) the standard terminology from machine learning [6], we call the algorithmic architectures that implement (at least approximately) optimal decision making *recognition models*. Although, in order to rigorously formalize the concept of uncertainty, we derived the definition of a recognition model based on a generative model, it is only the recognition model that needs to be actually implemented in the brain for decision making (though see [7] for potential uses and signatures of generative models also being implemented). A key question is then what recognition models the brain employs and specifically which, if any, of their internal variables are computed probabilistically, that is, such that uncertainty about their values is represented.

The conceptually most straightforward recognition model is broken down into two discrete steps that directly correspond to the observation and decision process components of the generative model: computing the posterior, and choosing the action (Figure 1, 3rd column). Indeed, these two steps have been suggested to correspond more broadly to the computations underlying perceptual (and memory [8]) processes, and action selection, respectively [9,10]. In this case, the representation of uncertainty is a given: the perceptual module computes a (potentially approximate) posterior probability distribution over the decision variable. Thus, such recognition models are *probabilistic*. However, note that, ultimately, the optimal action is just a function of the observations (Equation 2b). This mapping from observations to action could be implemented directly, without having to ever compute explicitly the posterior over the decision variable. In this case, even if the computation of the optimal action is broken down to several internal steps and quantities, none of these need to correspond to the decision variable as such, or its Bayesian posterior. Thus, the resulting recognition model is *non-probabilistic* (Figure 1, 7th column).

If optimal actions can be computed non-probabilistically, why bother with the costly computation of a posterior over the decision variable at all? Indeed, some of the most successful deep learning architectures of today do not use probabilistic representations [11]. Yet, there is widespread evidence for uncertainty about decision variables being represented in the brain [12,13,14^•^]. This suggests a normative reason for such probabilistic representations. Here we briefly review four such reasons, each of which implies that the additional computational cost of probabilistic representations can be offset by their increased data-efficiency and memory-efficiency (i.e. that they can perform well with less learning and require less memory):

Task-flexibility. The modular architecture of probabilistic recognition models endows them with great flexibility when a new task is encountered [15]. In this case, the ‘perception’ module of the recognition model can be kept the same, and only ‘action selection’ needs to be adapted to the new task by incorporating the corresponding new utility function into it. For example, if one day a car blocks our way to the right at the parking station, then the action of turning right suddenly yields very low utility no matter where our bike is parked. Nevertheless, with a probabilistic recognition model, we can keep using the same perceptual module to compute the relative location of the bike as we have always done. In contrast, an altogether new non-probabilistic recognition model would need to be constructed (or learned) whenever the utility function changes, precisely because non-probabilistic recognition models allow no neat splitting between perception and action selection.
Information fusion. When information from multiple observations needs to be fused, probabilistic recognition models for each observation can be combined efficiently. This is because the posterior (or, more precisely, the likelihood; Box 1) over the decision variable they compute is the ‘sufficient statistic’ of the corresponding observation. Information fusion is not only relevant for classical cases of (multi)sensory cue combination, when the different observations correspond to different sensory modalities [16,17] or cues [18] (e.g. depth cues for judging the distance of our bike) or memory [19] (as when combining our current percept of a bike that looks like ours in the distance and our memory of where we left our bike), but also for accumulating evidence across successive observations over time [20] (successive views of the parking station as we walk towards our bike). Optimal sensory cue combination can be achieved by combining the individual probabilistic recognition models appropriate for each sensory (or memory) observation [21], and optimal evidence accumulation can be performed by applying the same probabilistic recognition model recursively [22] (also known as Bayesian filtering [23]). In contrast, to achieve the same performance with non-probabilistic recognition models, different models need to be constructed (or trained) for each combination of sensory cues that may need to be combined in sensory cue combination. For evidence accumulation, they would need to have access to the entire relevant history of observations at once.
Active sensing. Information gathering is often an active rather than a passive process: we can control our sensors (e.g. move our eyes) so that we receive information that is most useful for solving a task (finding our bike). The Bayesian posterior over the decision variable, as computed by probabilistic recognition models, can be directly used to form an objective for active sensing [24^•^]. For example, active sensing can be achieved by actions whose predicted sensory consequences would leave the least amount of uncertainty about the decision variable (quantified by the entropy of the posterior), or by actions that would result in a posterior under which the expected utility (using the optimal action) is the highest [25]. Importantly, for computing the possible sensory consequences of these actions in both cases, the same generative model of decision making can be extended to include the effects of active-sensing-related actions on observations (Figure 1). Such a recycling of resources makes active sensing data-efficient. In contrast, information gathering in non-probabilistic recognition models implies an extension of the space of possible actions (e.g. in the case of our bike search, our actions become tuples consisting of which way to look *and* then turn) requiring to learn an entirely new recognition model for each active sensing action. Even worse, the available supervision for information gathering actions in this case is very sparse and indirect — we make many eye movements while searching for our bike, these eye movements by themselves are not rewarded, and each of them contributes only very indirectly to our ultimate success or failure to find the bike. In sum, achieving efficient active sensing with a non-probabilistic recognition model is often just altogether impractical.
Learning. In most cases, the recognition model can be improved with experience, such as learning to distinguish our bike from similar bikes often parked at the same station. However, this can be a non-trivial task without receiving ground-truth information. For example, if we accept occasionally a colleague's offer of a lift home while hesitating at the station about the direction to find our bike, we learn neither the true value of the decision variable (where the bike was), nor the correctness of the action we would have chosen (which way to go). Thus, there is no obvious way to learn from those occasions — as long as one uses a non-probabilistic recognition model. In contrast, by using a probabilistic recognition model, one can still take advantage of such ‘unlabelled’ data for improving the recognition model by averaging over the possible adjustments that would be appropriate for different values of the decision variable. Importantly, to weight appropriately the different terms in this average, the uncertainty about the decision variable (as quantified by its likelihood or the posterior, Box 1) needs to be taken into account. In other words, probabilistic recognition models are particularly well-suited for unsupervised or semi-supervised forms of learning.

There are also ways to interpolate between probabilistic and non-probabilistic models in an attempt to combine the best of both worlds: the data-efficiency and memory-efficiency of probabilistic models on one hand, and the computational efficiency and direct performance guarantees on the relevant decision tasks of non-probabilistic models on the other (Box 2).

Box 2 **Between probabilistic and non-probabilistic recognition models**

**Amortized inference**

Definition: Computational resources are shared across subsequent uses of the recognition model. In the context of Bayesian inference, this can be accomplished by treating latent variables as if they were observed, substituting a previously inferred value for them.
Machine learning application: Once a non-probabilistic deep neural network has been extensively trained on some tasks, it can be reused almost entirely (bar the final one or few layers) in novel but related tasks, such that it can achieve state-of-the-art performance already after only minimal training on those tasks [26].
Behavioral evidence: ‘Certainty-equivalent’ form of amortization has been shown to account for suboptimalities in high-level human probabilistic reasoning [27] as well as lower level ‘conditioned perception’ effects, whereby an earlier perceptual discrimination decision biases later perceptual estimates of the same variable [28].

**Loss-calibrated inference**

Definition: Rather than using general-purpose algorithms to speed up probabilistic inference, knowledge about the utility (or loss) function specific to the current task is used for setting up the particular approximate inference algorithm to be used.
Machine learning application: Loss-calibration increases the expected utility of decisions during variational inference [29].
Behavioral evidence: A particular form of loss-calibration, called ‘utility-weighted inference’, has been shown to account for a number of widely observed, seemingly irrational cognitive biases in human decision making, including the over-representation of extreme event probabilities [30].

Alt-text: Box 2

## Representing uncertainty in a complex world

Although the simplest two-step generative model described above succinctly summarizes the essential elements of Bayesian decision making (Figure 1, 1st column), it belies the real complexity of the natural environment. In most cases, our observations are generated by a complex mesh of interactions between a large number of latent variables, of which the decision variable is but one (Figure 1, 2nd column). For example, our perceived view of the parking station is jointly determined by a number of features characterizing each individual bike and car (their make, model, color, accessories, locations, etc.) as well as the lighting conditions and our viewing angle among other aspects.

In complex environments, of which the internal generative model includes multiple latent variables, probabilistic recognition models can be further subdivided based on how many of the latent variables they represent probabilistically. The conceptually most straightforward, but computationally most ambitious, recognition model is based on a direct, *fully Bayesian* inversion of the generative model (Figure 1, 4th column). In fully Bayesian recognition models, the (joint) posterior over all latent variables of the generative model implies a constitutive representation of uncertainty because uncertainty is represented for variables irrespective of whether they are directly relevant for the current decision making task (i.e. whether they are the designated decision variable). At the other — but still probabilistic — extreme, the recognition model only computes a posterior over the decision variable, but not over any of the other variables of the generative model (Figure 1, 6th column). Of course, there may still be multiple internal variables storing interim results of the computations of recognition, but those need not correspond to the latent variables of the generative model, and even if they do, no posterior needs to be computed over them at all (e.g. even when representing the color of each bike in a visual scene, only the single best estimate of each color is represented, rather than its full posterior.) Thus, in this case, the recognition model remains *task-dependent* as it represents uncertainty opportunistically only for the decision variable relevant for the current task. Finally, there also exists a continuum of intermediate, *hybrid* models between these two extremes: these models compute the posterior over, and thus represent uncertainty about only a subset of latent variables but not all of them (Figure 1, 5th column).

Note that the benefits of probabilistic recognition models enumerated in the previous section already apply to even the least ambitious of these models, the task-dependent recognition model. Meanwhile, the computational complexity of inference in a fully Bayesian recognition model grows exponentially with the number of latent variables in the general case. Thus, extending our previous discussion, we can ask why bother with the costly computation of a joint posterior over all (or a large number of) the latent variables? Indeed, the training of deep neural networks, for example for image classification, usually requires uncertainty to be correctly represented only in the decision variable (image class label) — if at all — and can still lead to human-level or even super human-level performance [31]. However, there are indications that the brain may be closer to the fully Bayesian model and represents uncertainty about sensory as well other variables, and not just the ones related directly to the decision [3]. The normative reasons for following this strategy are based on the same general trade-off between computational costs and data-efficiency and memory-efficiency that we argued underlay the benefits of probabilistic recognition models:

Task-flexibility. Just as different tasks may differ in their utility function over a particular latent variable, they may also differ in the identity of the latent variables (or subsets of latent variables) upon which the utility function depends. For example, it is only in the context of the specific task of locating our bike that out of all the latent variables characterising the parking station, it is the location of our bike that happens to play the special role of the decision variable. Once we find our bike and cycle through the station, suddenly other variables (the location of the exit, the predicted trajectory of an approaching car, etc.) would subsume the status of the decision variable for choosing our actions (which way to turn the handle bar). In such cases, when using task-dependent recognition models, separate models need to be constructed for each of these tasks. A fully Bayesian recognition model is much more economical in that perception can proceed unchanged, as it constitutively computes the posterior distribution over all the latent variables, and only action selection needs to be modified to reflect the new utility function.
Information fusion. As we argued above, efficient information fusion across different observations (as in sensory cue combination or evidence accumulation) requires probabilistic representations. Importantly, this efficiency of probabilistic recognition models is only guaranteed as long as they represent uncertainty about a sufficiently large set of variables. This is because the computations underlying cue combination and evidence accumulation can only be performed efficiently (by a simple combination of individual recognition models for cue combination, and by simple recursive operations, one observation at a time, for evidence accumulation) if different observations are statistically independent given the latent variables about which uncertainty is represented [32]. However, in complex environments, observations are rarely dependent only on the single latent variable that happens to determine the utility of our actions (i.e. the decision variable). For example, two subsequent views of the parking station (the observations) across which we need to fuse information to better infer the location of the bike (the decision variable), will not be statistically independent given the location of our bike, because there are a number of other latent factors which influence both views (the location of other bikes, lighting conditions, etc.). In turn, conditioning on all these latent variables can make the observations independent. (Sidenote: this is closely related to the problem of what constitutes an appropriate representation of ‘state’ in sequential decision making tasks, which are an extension of the non-sequential decision making tasks we are considering here, and which can be formalized as Markov decision processes in the realm of reinforcement learning [33].) Thus, in these cases, only recognition models that are sufficiently close to being fully Bayesian will be able to fuse information efficiently. In contrast, task-dependent recognition models, in which only the decision variable is represented probabilistically, inherit the same problems that we argued non-probabilistic recognition models have: information fusion requires a separate recognition model for each combination of cues, or access to a history of observations.
The advantage of inferring multiple latent variables jointly is particularly well exposed in tasks that require ‘explaining away’: a special form of credit assignment in updating beliefs when an additional observation leads to drastic changes in the posterior. Such updating occurs when the additional observation reveals that previous observations that have been attributed to a particular cause should in fact be credited to an entirely different cause. A classical example for this is when an object that initially looks convex based on its shading suddenly looks concave when we receive evidence that it is actually lit from below not above [34]. This can be implemented naturally in a recognition model that jointly infers the shape of the object and the direction of light.
Active sensing. Generally, active sensing is a sequential process: it takes multiple adjustments of our sensors to collect adequate information before making a decision. For example, when looking for our bike at the parking station, we move our gaze to several locations to reduce our uncertainty about the location of our bike before we decide which way we turn. Merging information across consecutive observations is precisely the problem of information fusion we discussed above. Therefore, the very same arguments explain why fully Bayesian representations are useful for active sensing in complex environments.
Learning. Formally, learning is ‘just’ another type of information fusion, albeit on a slower time scale than what we considered so far. Instead of fusing information across multiple observations within a single trial to update the decision variable or other latent variables (as in evidence accumulation), learning requires fusing information across multiple trials to update the recognition model itself. Therefore, the same arguments that justify representing uncertainty about latent variables in a recognition model also apply to representing uncertainty about the recognition model itself (its parameters, structure, or form [35]): efficient online learning requires probabilistic representations. As in all these cases uncertainty needs to be represented about quantities (parameters, etc.) in the recognition model other than the decision variable, such recognition models are not task-dependent any more by our definition, and are closer to the fully Bayesian end of the spectrum. For example, in neural networks, a probabilistic representations of synaptic weights (the parameters of the recognition model) has been shown to be advantageous as it allows optimal adaptation of learning rates on novel tasks [36^•^] and helps avoiding catastrophic forgetting across multiple tasks [37].
Moreover, just as representing uncertainty about the decision variable (and other variables, as we saw above) can allow efficient information gathering in active sensing, representing uncertainty about the recognition model can allow efficient information gathering for active learning, that is, to choose inputs that are expected to improve the future performance of the recognition model most [25]. On the first occasion when we search for our bike at the parking station, we might choose a direction that leads to a longer expected searching time than the optimal choice, and use the extra time to familiarize ourselves with the station so that to improve our recognition model and thus our future search performance. Representing uncertainty about the recognition model can help us focus our exploration of the station where we know least about it. Eventually, once we have little uncertainty left about it, we can decide to stop exploring altogether.

## Implementing probabilistic recognition models in the brain

Whether probabilistic recognition models in perception are task-dependent, fully Bayesian, or hybrid, has important implications for how they might be implemented in the brain. For example, at the broadest level, fully-Bayesian recognition models must maintain a globally coherent representation of their latent variables’ joint posterior. This requires that information about higher level cognitive variables should have effects on inferences about lower-level variables, that is, potentially strong top-down influences on sensory cortical areas [54]. There is a large swathe of experimental data on such top-down interactions [55]. More specifically, recent studies provided evidence for trial-specific priors, cued by auditory stimuli, affecting both overt decisions and early visual cortical responses in a visual perceptual decision making task [56,57]. While task-dependent models may not be formally incompatible with such top-down influences, they also do not make any specific prediction about them.

In the following, for a finer level of distinction, we review previous specific suggestions for how the brain might represent uncertainty, and group these representations by the kind of recognition models they may be able to implement. In Table 1, we also provide pointers to some of the key empirical data that have been suggested to support them. We note, however, that most of these data only provide circumstantial evidence for the corresponding representations as yet. Thus, more work will be necessary that directly contrasts the predictions of different representations and compares them to experimental data [53^••^].

**Table 1:** Compatibility of specific implementations of probabilistic recognition models with behavioral and neural data

recognition model	implementation	behavioral data	neural data
task-dependent	DDM	psychometric and chronometric curves of perceptual decisions [38,39]	decision-related ramping activity of LIP single cells [38, 39, 40]
	PPC	psychometric and chronometric curves of perceptual decisions [22]; cue combination (qualitatively) [21]	Poisson-like variability of cortical neurons [21]; single cell activity in LIP [22]
	DNN	object recognition and categorization performance [11,41]	feature selectivity along the hierarchy of visual cortex [42]

hybrid	CRP	human categorization [43]	–

fully Bayesian	belief propagation	bistable perception [44]; hallucinations [45]	tight balance between excitation and inhibition [46]
	extended PPC	–	anatomy and physiology of the olfactory bulb [47]
	DDC	–	dopaminergic or hippocampal activity [48]
	sampling	cue combination [18]; multistable perception [18,49]	various static [50, 51, 52] and dynamic [53] activity patterns of the early visual cortex

Details of the specific models are discussed in the main text. As we also note there, implementations appropriate for fully Bayesian recognition models could also implement task-dependent recognition models (but not vice versa). Thus, data listed here as supporting such implementations (e.g. multistable perception for sampling) does not necessarily provide support for fully Bayesian recognition models *per se*. Experiments providing support for fully Bayesian recognition models are discussed later (see also Table 2).

There have been three influential proposals for how task-dependent recognition models might be implemented in the brain (Figure 1, 6th column): the drift diffusion model (DDM [38]), probabilistic population codes (PPCs [21]), and deep neural networks (DNNs [42,41]). The DDM is a psychological process-level model of decision making, of which the behavioral signatures and neural underpinning have been extensively investigated (Table 1). According to the DDM, decisions are based on gradually accumulating noisy evidence obtained from sensory inputs [39] or memory traces [58]. Optimal decision making requires both the accumulated evidence and the time elapsed since the beginning of accumulation in such tasks [39], such that these two quantities together form the (sufficient statistic of the) ‘decision variable’, z, in our formulation (Figure 1). Importantly, the evidence accumulated by the DDM is always about a specific decision variable that is relevant for the current task (e.g. saccade left or right). Therefore, the DDM is a *bona fide* task-dependent probabilistic recognition model.

According to probabilistic population codes (PPCs), neural populations encode probabilistic information in a format that is both easy to read out by downstream areas and allows simple, biologically plausible neural operations (e.g. linear summation of inputs) to implement probabilistically optimal processing of such information [21,22]. As some PPCs were specifically designed to capture hierarchical probabilistic computations for example in a cue combination task [21], they might superficially appear to be fully Bayesian. Indeed, the neural architecture of such PPCs typically consists of several (feed-forward connected) layers, each encoding probabilistic information. Nevertheless, in our classification, they implement task-dependent recognition models because, at least in their originally proposed form [21], all layers encode probabilistic information about the same single decision variable (its likelihood based on different subsets of observed variables).

Recently, the most popular recognition models have been deep neural networks (DNNs). DNNs are typically trained on a given task in an end-to-end fashion by providing (a typically large set of) example input-output pairs from which these architectures can learn to generalize and generate the correct output to novel inputs. As such, DNNs are often used — and certainly construed — as fundamentally non-probabilistic recognition models that generate their output (the equivalent of a in our terminology) without performing any probabilistic computations on the way. Nevertheless, when trained with the appropriate (cross-entropy based) loss function, routinely used for example in image classification tasks, neural activities in the layer before a (the last hidden layer) come to essentially encode the posterior distribution of the decision variable z. As this probabilistic representation only emerges at this final stage, these DNNs are task-dependent probabilistic recognition models. In addition, even the probabilistic representation of z can be poorly calibrated in these models, as has been demonstrated, for example, with so-called ‘adversarial samples’ [59]. We posit that this lack of proper calibration in the last hidden layer might be the consequence of the missing representations of uncertainty in the intermediate layers. Indeed, it has been suggested that endowing DNNs with more fully Bayesian probabilistic representations (e.g. by dropout) might improve their calibration of uncertainty on adversarial samples [60^•^].

Regarding fully Bayesian recognition models (Figure 1, 4th column), there have been two different classes of neural representations suggested. In principle, each of these neural representations is also able to support task-dependent recognition models, but the differences between them are best exposed when applied to fully Bayesian recognition models. In the first such representation, neural activities represent parameters or sufficient statistics of the posterior over *all* relevant latent variables. One recent example of this class is distributed distributional codes (DDCs, [61^•^]). DDCs have been shown to have a number of computationally appealing properties, in particular when the recognition model corresponds to a complex hierarchical generative model (i.e. the very setting which motivates fully Bayesian recognition models in the first place, Figure 1, 2nd column) and needs to be learned in an unsupervised way from experience.

Other examples of parametric neural representations do not attempt to represent the full joint posterior over all latent variables and instead use a cruder factorized approximation, in which only the marginal posteriors over individual latent variables are represented [62,46,47]. Although such ‘marginally’ fully Bayesian recognition models lose all information about posterior correlations between latent variables, this simplification also greatly reduces the complexity of neural dynamics required to implement them based on methods borrowed from machine learning, such as belief propagation [62,46] or more general variational approximation schemes [47]. Some models in this class can be seen as extensions of PPCs to the (marginally) fully Bayesian case [62,63], inheriting some of their appealing properties, albeit with substantially more complex neural dynamics.

The other class of fully Bayesian recognition models uses a sampling-based representation of uncertainty [64,12]. In these models, neural responses represent the latent variables themselves such that the distribution of neural responses generated by the network's dynamics over some time period represents the joint posterior distribution of the recognition model. As such, these models again approximate the full joint posterior over all latent variables. There is substantial converging behavioral and neural evidence for sampling-based fully Bayesian recognition models at least in the early visual cortex (Table 1). Nevertheless, it remains to be seen whether such models apply to other perceptual domains and brain areas. This will be particularly interesting in settings in which inference over dynamically changing variables needs to be performed, as the time requirements of sampling may produce unique testable predictions in these domains [65].

There is one specific domain where hybrid recognition models (Figure 1, 5th column) have — by necessity — been proposed in cognitive science. When the generative model includes an infinite number of latent variables, Bayesian inference necessarily needs to focus on a finite subset of these to tractably compute the posterior. This is the realm of non-parametric Bayesian inference in machine learning [66]. Such non-parametric Bayesian models have been suggested to underlie a number of cognitive processes [67]. For example, a non-parametric Bayesian inference algorithm (called the ‘Chinese restaurant process’, CRP) can be used to infer which out of a potentially infinite number of categories does each item in a training set belongs to, and what the common characteristics of items in each category are [68]. Nevertheless, a systematic exploration of hybrid recognition models has not been pursued in cognitive neuroscience. This could be an interesting avenue for future research because these models have the potential to achieve a useful balance between performance and efficiency.

## Behavioral evidence for fully Bayesian recognition models

The top row of Table 2 summarizes the four normative advantages of probabilistic recognition models over non-probabilistic ones that we discussed above and show how these advantages translate into experimental designs that distinguish between these classes. Our approach is based on the superior generalization properties (high data and memory efficiency) of probabilistic recognition models. The critical insight is that while non-probabilistic recognition models can also learn to solve any decision making task, they can only do so after sufficiently long training. Thus, a proper test of probabilistic recognition models must create a situation in which the data- (or memory)-inefficient strategy of non-probabilistic models would be pushed to its limits. The strongest experimental tests capitalize on the extreme data efficiency of probabilistic models allowing one-shot generalization, previously investigated under the rubric of ‘Bayesian transfer’ [77]. In addition, the top row of Table 2 lists other, more subtle experimental tests that can still provide supporting evidence, based on the data and memory efficiency of probabilistic recognition models. While there have been previous proposals for the criteria that such experiments must meet [77,4], our approach based on normative principles allowed us to extend these proposals to other kinds of experiments that had not been considered in this context before.

**Table 2:** Behavioral evidence for probabilistic and fully Bayesian recognition models

	normative advantages			
recognition model	task-flexibility	information fusion	active sensing	learning
probabilistic versus non-probabilistic	[69,70]	[19,65,71^•^,^72^]	[24^•^,^73^]	[74^•^75]
fully Bayesian versus task-dependent	[65,76^••^]	–	–	–

Studies providing behavioral support that the brain's recognition model is probabilistic (top row) or fully-Bayesian (bottom row) are organized according to the normative advantage of the probabilistic or fully Bayesian recognition model they verified behaviorally (columns). Below we summarize the evidence for probabilistic recognition models only, the existing evidence (or lack thereof) for fully-Bayesian recognition models is discussed in the main text. **Task-flexibility.** Humans (or monkeys) were found to exhibit a high degree of task-flexibility in tasks requiring generalization either to new utility functions [69] or to new stimuli giving rise to posterior distributions that are qualitatively different from those previously experienced in the task [70]. **Information fusion.** Studies of information fusion showed that humans can near optimally combine two sources of information. Notably, in several classical cue-combination studies, participants had plenty of everyday experience with combining the information from the two tested sensory modalities to enhance the absolute accuracy of their decisions [16,17]. Thus, these tasks required only modest generalization, and as such could be solved with non-probabilistic recognition models. Other studies showed optimal information fusion even when participants needed to combine two sources of information that they had never combined before [19], or a sequence of observations while the number and informativeness of observations was varied [71^•^]. In these cases, having a separate (non-probabilistic) recognition model for each task condition would be infeasible. Moreover, humans provided reliable uncertainty reports about their own performance when the difficulty of trials was modulated by multiple task parameters (e.g. the number and contrast of items in a scene) [65], suggesting that uncertainty reports were based on a unified representation of uncertainty (i.e. a single posterior distribution) rather than heuristic estimates corresponding to the different task parameters. Furthermore, in line with a unified uncertainty representation, the reported uncertainties also reliably predicted stimulus-independent fluctuations in performance over and above those controlled by experimentally defined cues [72]; Á Koblinger *et al.*, 2019, COSYNE, conference]. **Active sensing.** Eye movements are almost never rewarded directly as such, but typically depend on participants’ inferences about the currently viewed stimulus. Thus, they offer an ideal test bed for assessing behavioral signatures of probabilistic representations. For example, while performing the same visual search task in widely different lighting conditions, humans near-optimally adjusted their eye-movements to the changed lighting conditions, suggesting that they could efficiently generalize their eye-movement strategies across a wide range of posteriors [73]. In a visual pattern categorization task, eye movements were also shown to be optimized for information search [24^•^]. Critically, this eye movement strategy correctly took into account the constantly evolving posterior distribution that a probabilistic recognition model computed over pattern category (the decision variable) based on (the growing set of) previous fixations in a trial (observations). **Learning.** As non-probabilistic recognition models provide no principled basis for unsupervised learning, appropriate stimulus reliability-dependent updating of a recognition model can be taken as a hallmark of the recognition model being probabilistic. Such optimal updating was reported in a perceptual discrimination task without feedback, in which human participants used their uncertainty estimates about the stimulus (the decision variable) to correctly update their estimate of the base rate of the stimulus (a parameter of the recognition model) [74^•^]. Similarly, in an economic decision task [75], participants near-optimally adjusted the learning speed of a dynamically fluctuating reward rate (decision variable), despite a lack of direct feedback about reward rates.

Analogously to the probabilistic versus non-probabilistic distinction, we can also use the normative advantages of fully Bayesian versus task-dependent recognition models to suggest experimental strategies for distinguishing these in behavioral measurements (Table 2, bottom row). As a disclaimer, we note that the experimenter can never have perfect knowledge about which latent variables constitute the internal generative model of the subject, and therefore, behavioral tests are insufficient to distinguish between fully Bayesian and hybrid recognition models. Nevertheless, by demonstrating that participants represent the uncertainty of latent variables other than the decision variable, one may be able to exclude task-dependent recognition models.

In general, there is a basic experimental criterion that needs to be met regardless of the specific normative advantage we aim to utilize: we need to use complex stimuli that are characterized by multiple latent variables that differ in the level of (un)certainty with which they can be inferred. Without this across-variable diversity in uncertainty, one cannot exclude the possibility that participants summarize the uncertainty of the whole stimulus in a single value.

Task-flexibility. Given the constitutive uncertainty representation of a fully Bayesian recognition model, it can rapidly switch between utility functions that treat different latent variables as the decision variables. This can be tested in a sequential manner by making each latent variable the decision variable, one-by-one, by changing the utility function of the task across the trials. There is, however, a caveat to this method, that once the identity of the decision variable is revealed, a task-dependent recognition model is sufficient to solve the task. Nevertheless, this problem can be eliminated by revealing the identity of the decision variable only after the stimulus presentation, a typical strategy in multi-item working memory tasks [78]. Multi-item working memory tasks represent a special case of this approach in which separate latent variables correspond to distinct items in a multi-element visual scene, and the identity of the queried item is only revealed once the stimulus disappears (typically after a delay period). Despite the widespread use of working memory experiments, only a small fraction of them is appropriate for identifying probabilistic recognition models at all [65,72,76^••^], and even within this smaller set of studies, we are only aware of two which were appropriate for distinguishing fully Bayesian from task-dependent recognition models [65,76^••^]. In these studies, the uncertainties associated with different items within the same scene were systematically varied by their contrast [65] or by an extraneous attentional cue [76^••^]. The representation of uncertainty about the queried item was assessed directly from participants’ uncertainty reports [65,76^••^], or indirectly from their categorization decisions (with non-trivial category boundaries, thus requiring an appropriate representation of uncertainty about stimulus orientation) [76^••^]. Both studies provided evidence for participants’ simultaneous representation of probabilistic information about multiple items in a scene.
The main drawback of the method used in typical working memory experiments is that it tests the (probabilistic) representation of latent variables (items in scene) one-by-one. Therefore, it can only provide evidence for the representation of the marginal posterior distributions of individual latent variables, not a full joint posterior over all of them. Testing the representation of joint posteriors would require complex utility functions that depend on more than a single latent variable. Spatial tasks, in which latent variables correspond to different spatial dimensions rather than different items, seem a natural choice for this. Indeed, humans have been shown to be able to integrate their uncertainty with complex utility functions in such tasks [79]. We suggest that adapting this approach to the study of fully Bayesian recognition models is a promising avenue for future research.
Information fusion. An important advantage of fully Bayesian recognition models over task-dependent ones is that they can efficiently fuse information across observations that are not independent given the decision variable. In order to experimentally test this, there need to be decision variable-independent correlations among observations due to additional latent variables in the task's generative model. In the domain of motor control, it has been argued that not only the state of the environment (decision variable) but also that of the body (additional latent variable) determine motor errors (observations) [80]. Critically, just as the state of the environment changes continually, so does the state of the body, thus creating correlations across the sequence of motor errors that we experience, requiring a joint probabilistic representation of environmental and body state for optimal behavior. Indeed, a fully Bayesian recognition model jointly inferring both latent variables successfully accounted for behavior in a variety of motor adaptation experiments [80,81]. Nevertheless, without explicitly investigating how well alternative models might be able to fit the data, the possibility of task-dependent recognition models cannot be fully excluded.
Interestingly, this kind of information fusion has not been exploited more generally to test for fully Bayesian recognition models. We suggest that future experiments could investigate information fusion in paradigms in which the decision variable (e.g. the color of a different object on each trial) needs to be estimated (e.g. based on observations of reflected light from the surface of the object) in the presence of non-decision (‘nuisance’) latent variables that have predictable temporal correlation structure across trials (mimicking, e.g. slowly changing lighting conditions across the day). A key manipulation would be providing extra information about the nuisance variable (e.g. by revealing the color of the lighting source) on some trials. Solving such tasks successfully requires both dynamical inference over the nuisance variable and explaining away between the nuisance and decision variables, which taken together implies performing joint inference over both variables — something that a task-dependent recognition model only inferring the decision variable could not achieve.
Active sensing. Just as in the case of passive information fusion, there can also be decision variable-independent co-variation across actively selected observations, which in turn can depend on nuisance variables. If these correlations modulate the informativeness of observations about the decision variable, then the active control of the sensors (presumably optimizing information about the decision variable) can benefit from representing the uncertainty of these nuisance variables. Although, once again, we are not aware of using such an active sensing approach to studying fully Bayesian recognition models, we suggest that it could be a fruitful future research direction. For example, the visual pattern categorization experiment used to study active sensing by Ref. [24^•^] (described in Table 2), could be extended such that correlations across observations (pixels of an image) not only depend on the decision variable (stripy versus patchy pattern, respectively defining the fall-off of spatial correlations between pixels to be longer in one direction than the other, or to be isotropic) but also on a nuisance variable (e.g. wavelength or spatial scale) that is not directly relevant for the task, but still influences correlations among observations. In this case, active sensing eye movements can benefit from inferring this nuisance variable (together with the decision variable), and this benefit should lead to behaviorally identifiable signatures.
Learning. As we saw previously, when learning needs to proceed unsupervised, the optimal adjustment of model parameters depends on the posterior distribution of the decision variable. The more accurately the recognition model approximates this posterior distribution, the more efficient learning will be. Thus, the efficient information fusion of fully Bayesian recognition models that improves inferences about the decision variable by also representing uncertainty about other latent variables (see above) should also improve unsupervised learning. Once again, this advantage of fully Bayesian recognition models has not been used to design specific experiments, although it could potentially reveal deep connections between the representation of uncertainty and learning.

## Conclusion

In contrast to distinguishing probabilistic from non-probabilistic recognition models (Table 2, top), there is a notable paucity of behavioral experiments studying the fully Bayesian versus task-dependent distinction (Table 2, bottom). This is not surprising given that this distinction has so far attracted little attention even at a conceptual level. The goal of this review was precisely to fill this gap.

First, we discussed a spectrum of possible recognition models with different uncertainty representations that can all compute optimal decisions in a given task, albeit at very different computational, data and memory costs. We argued that in this respect, fully Bayesian recognition models stand out with their superior data and memory efficiency, which allows for efficient generalization across a wide range of tasks and observation conditions. Given the parsimony of the hypothesis, it seems appealing to assume that general-purpose human and animal brains implement fully Bayesian recognition models in order to flexibly use the limited amount of experience they may have with any one task. Although there are neurally plausible implementations of fully Bayesian recognition models that can explain a number of neurophysiological observations, the available evidence is not conclusive and, ultimately, behavioral evidence will also be necessary to establish whether the recognition models the brain implements are task-dependent or closer to being fully Bayesian. Therefore, in this paper, we organized the normative benefits of probabilistic and, more specifically, fully Bayesian recognition models from the perspective of the key cognitive advantages they offer (task-flexibility, information fusion, active sensing, learning). This allowed us to establish a set of experimental criteria that is suitable for distinguishing task-dependent and fully Bayesian (or hybrid) recognition models. We then reevaluated existing experiments and data in the light of these criteria and, finally, made constructive suggestions for new paradigms that could be used in future behavioral experiments. Our hope is that these ideas will provide a useful guide for future investigations into the nature of probabilistic representations in the brain — one of the most fundamental questions linking neural computation and behavior.

## Conflict of interest statement

Nothing declared.

## References and recommended reading

Papers of particular interest, published within the period of review, have been highlighted as:

• of special interest
•• of outstanding interest
